# ApoA-I Mimetic Peptide Reduces Vascular and White Matter Damage After Stroke in Type-2 Diabetic Mice

**DOI:** 10.3389/fnins.2019.01127

**Published:** 2019-10-25

**Authors:** Xiaohui Wang, Rongwen Li, Alex Zacharek, Julie Landschoot-Ward, Michael Chopp, Jieli Chen, Xu Cui

**Affiliations:** ^1^Department of Neurology, Henry Ford Hospital, Detroit, MI, United States; ^2^Department of Physics, Oakland University, Rochester, MI, United States

**Keywords:** diabetes, stroke, blood–brain barrier (BBB), white matter (WM), inflammation

## Abstract

Diabetes leads to an elevated risk of stroke and worse functional outcome compared to the general population. We investigate whether L-4F, an economical ApoA-I mimetic peptide, reduces neurovascular and white-matter damage in db/db type-2 diabetic (T2DM) stroke mice. L-4F (16 mg/kg, subcutaneously administered initially 2 h after stroke and subsequently daily for 4 days) reduced hemorrhagic transformation, decreased infarct-volume and mortality, and treated mice exhibited increased cerebral arteriole diameter and smooth muscle cell number, decreased blood-brain barrier leakage and white-matter damage in the ischemic brain as well as improved neurological functional outcome after stroke compared with vehicle-control T2DM mice (*p* < 0.05, *n* = 11/group). Moreover, administration of L-4F mitigated macrophage infiltration, and reduced the level of proinflammatory mediators tumor necrosis factor alpha (TNFα), high-mobility group box-1 (HMGB-1)/advanced glycation end-product receptor (RAGE) and plasminogen activator inhibitor-1 (PAI-1) in the ischemic brain in T2DM mice (*p* < 0.05, *n* = 6/group). *In vitro*, L-4F treatment did not increase capillary-like tube formation in mouse-brain endothelial cells, but increased primary artery explant cell migration derived from C57BL/6-aorta 1 day after middle cerebral artery occlusion (MCAo), and enhanced neurite-outgrowth after 2 h of oxygen-glucose deprivation and axonal-outgrowth in primary cortical neurons derived from the C57BL/6-embryos subjected to high-glucose condition. This study suggests that early treatment with L-4F provides a potential strategy to reduce neuroinflammation and vascular and white-matter damage in the T2DM stroke population.

## Introduction

Diabetes mellitus (DM) is a major risk factor for ischemic and hemorrhagic stroke. Stroke patients with DM exhibit a worse neurovascular prognosis and white-matter (WM) lesion and neurological function than non-DM stroke patients. Therefore, development of therapeutic approaches for the DM-stroke population are urgently needed.

Type-2 diabetes (T2DM) constitutes 90% of the DM population. DM patients have low levels of blood high-density lipoprotein (HDL) (Maron, 2000; Sharrett et al., 2001; Gotto and Brinton, 2004; Bonora, 2006) and impairment of HDL function such as the antioxidative capacity of HDL (Bonora, 2006; Kontush and Chapman, 2006; Anan et al., 2010; Kruit et al., 2010; Mulder et al., 2011; Scheffer et al., 2011; Dullaart et al., 2012). Improved HDL functionality may contribute to maintenance of pancreas beta-cell function in subjects with well-controlled T2DM (Dullaart et al., 2012). Impairment of HDL anti-oxidative function in T2DM contributes to enhanced formation of oxidative stress products, such as advanced glycation end products (AGEs) (Mulder et al., 2011; Scheffer et al., 2011). Dysfunction of HDL also promotes inflammatory effects (Kruit et al., 2010) and increases proinflammatory factor activation, such as tumor necrosis factor alpha (TNFα) (Igarashi et al., 2008; Nayak et al., 2010; Gomez-Banoy et al., 2016), high-mobility group box 1 (HMGB1) and AGE receptor (RAGE) (Williams and Nadler, 2007; Ye et al., 2011), and plasminogen activator inhibitor-1 (PAI-1) (Scarabin et al., 1998; Bonora, 2006; Williams and Nadler, 2007; Al-Hamodi et al., 2011; Ye et al., 2011; Costa and Soares, 2013; Gorska-Ciebiada et al., 2016), which can exacerbate vascular and WM damage after stroke in T2DM. Therefore, improving HDL function through modification of its lipid and/or protein content maybe a therapeutic target for the treatment of stroke in T2DM.

In human, the circulating blood contains only about 40% of the total amount of HDL, with most of HDL particles present in human interstitial fluids including the arterial intimal fluid (Miller et al., 2013). The HDL particles in plasma contain either a single copy or multiple copies of apolipoprotein A-I (ApoA-I). ApoA-I has been shown to possess several atheroprotective functions, including inhibition of inflammation. However, ApoA-I is a selective target for oxidation by myeloperoxidase, which results in impaired HDL function (Imaizumi et al., 2011). 4F is an economical 18 amino acid peptide mimetic the tertiary structure of ApoA-I. There are two 4F species, D-4F (right hand) and L-4F (left hand). When administered subcutaneously, D-4F and L-4F are equally efficacious, D-4F is orally efficacious but L-4F is digested by gut proteases (Navab et al., 2009). Both of them have remarkable anti-inflammatory properties based on their ability to preferentially bind proinflammatory oxidized lipids and decrease serum oxidized low-density lipoprotein (LDL) level (Van Lenten et al., 2008; Liu et al., 2014; Yao et al., 2015), improve HDL function (Ou et al., 2005; Van Lenten et al., 2008; Yu et al., 2008; Navab et al., 2009; Nandedkar et al., 2011; Qin et al., 2012), and increase cholesterol efflux (Navab et al., 2004; Sherman et al., 2010). Our previous studies show that D-4F treatment of wild-type mice subjected to stroke reduces vascular and WM damage as well as improves recovery of neurological function 14 days after stroke (Cui et al., 2016). Rats with type-1 diabetes and subjected to stroke receiving orally administered D-4F initiated at 2 h after stroke, and subsequently at 24 and 48 h after stroke exhibit significantly decreased neuroinflammation (Ning et al., 2017). However, to our knowledge, there are no studies investigating whether L-4F has a therapeutic effect on stroke-induced vascular and WM damage in T2DM mice. In this study, we therefore investigated the hypothesis that anti-inflammation may play an important role in L-4F treatment of stroke induced neuroprotection and reduction of WM damage in a clinically relevant stroke model using db/db T2DM mice.

## Materials and Methods

### Animal Stroke Model and Experimental Groups

The number of animals for the *in vivo* study was calculated *a priori* by power analysis. For blood biochemistry, lesion volume, and histochemical/immunohisto-staining measurement, 11 mice per group survival stroke animals were targeted to achieve a power of 0.83 at a significance level of < 0.05, assuming 25% difference in mean, a 20% standard deviation at the 95% confidence level. For Western-blot (WB) and real time-quantitative PCR (RT-qPCR) assays, 6 stroke mice per group were needed. To meet these experimental targets, a total of 90 adult male T2DM mice (BKS.Cg-*m* + / + *Lepr^*db*^/J*, db/db mice, 3 month old, Jackson Laboratory, Wilmington, MA, United States) were used. For middle cerebral artery occlusion (MCAo) surgery, animals were anesthetized with 2% isoflorane in a jar for pre-anesthetic, and spontaneously respired with 1.5% isoflurane in 2:1 N_2_O:O_2_ mixture using a facemask connected and regulated with a modified FLUOTEC 3 Vaporizer (Fraser Harlake). Rectal temperature was maintained at 37°C throughout the surgical procedure using a feedback regulated water heating system. Transient right MCAo was induced for 1 h by advancing a 6-0 surgical nylon suture (8.0–9.0 mm), determined by the animal weight, with its tip rounded by heating near a flame, to block the origin of the MCA, using a method of intraluminal vascular occlusion modified in our laboratory (Liu et al., 2007). One hour after MCAo, reperfusion was performed by withdrawal of the suture. Two hours after suture withdrawal mice were randomly separated into three groups by drawing different colored balls:

(1) Sham-control group: mice were subjected to the same procedures as the MCAo without insertion of filament (*n* = 4 mice).
(2) MCAo group: mice were subcutaneously administered saline daily for 4 days (*n* = 52 mice).
(3) L-4F treatment group: mice were administered L-4F (BioMatik, Cambridge, ON, Canada) 16 mg/kg (*n* = 34 mice) and subsequently daily for 4 days. All survival animals were sacrificed 4 days after MCAo.

### Functional Tests

To evaluate neurological functional deficits and recovery after stroke, all animals were evaluated on the modified neurological severity score (mNSS, the total score is 12) and left foot-fault test before MCAo (as the baseline) and at 1, 3, and 4 days after MCAo, as previously described (Chen et al., 2001; Shehadah et al., 2014). Functional analyses were performed by an investigator blinded to the experimental groups.

### Blood Biochemistry Measurement

To test blood biochemistry, the animals were fasted overnight and blood was collected from tail vein before MCAo as the baseline and prior to sacrifice. Blood levels of glucose were measured using glucose test strips in a glucose analyzer (Accu-Chek Compact System; Roche Diagnostics, Basel, Switzerland), and the levels of HDL, total-cholesterol (T-CH) and triglyceride were tested using CardioChek P•A analyzer (Polymer Technology System, Inc., Indianapolis, IN, United States), following the manufacturer’s instructions. Each sample was tested in triplicate and the data are presented as mg/dl values.

### Cerebral Hemorrhagic Transformation, Lesion Volume, and Survival Rate Measurement

All brains were fixed by transcardial-perfusion with saline, followed by perfusion and immersion in 4% paraformaldehyde and were then embedded in paraffin. Using a mouse brain matrix (Activational Systems Inc., Warren, MI, United States), the cerebral tissues were cut into seven equally spaced (1 mm) coronal blocks, and a series of adjacent 6 μm thick sections were cut from each block. Seven coronal sections of tissue were processed and stained with hematoxylin and eosin (HE). For calculation of brain hemorrhage volume, the percentage areas of petechial and gross hemorrhage were measured in each histological section and summed. For lesion volume measurement, the indirect lesion area was calculated, in which the intact area of the ipsilateral hemisphere was subtracted from the area of the contralateral hemisphere. Lesion volume is presented as a volume percentage of the lesion compared with the contralateral hemisphere (Swanson et al., 1990). For evaluation of mortality, all animals were counted daily. The total number of dead animals in each group was counted within the 4 days after MCAo. The survival rate is presented as a percentage of the total number of stroke animals in each group.

### Histochemical and Immuno-Staining

For histochemical/immunostaining, a standard paraffin block was obtained from the center of the lesion (bregma −1 to +1 mm). A series of 6-μm thick sections were cut from the block. Every 10^*th*^ coronal section for a total of five sections was used. Histochemical-staining for Bielshowsky silver (BS, an axon marker) and Luxol fast blue (LFB, a myelin marker), or histoimmino-staining for antibodies against albumin (BBB leakage marker, 1:500; Abcam), von Willebrand Factor (vWF, a vessel marker, 1:400; Dako), α-smooth muscle actin (αSMA, a smooth muscle cell-SMC marker, 1:800, Dako), SMI31 (a marker of phosphorylated-neurofilament, 1:1000, Covance), platelet-derived growth factor receptor alpha (PDGFRα, a marker of oligodendrocyte progenitor cells-OPCs, 1:100, Chemicon), and HMGB1 (1:800, Abcam) were performed. For immunostaining measurement, five sections with each section containing 8 fields of view within the cortex and striatum from the ischemic boundary zone (IBZ), defined as the area surrounding the lesion, which morphologically differs from the surrounding normal tissue, were digitized using a 40X objective (Olympus BX40) using a 3-CCD color video camera (Sony DXC-970MD) interfaced with an MCID computer imaging analysis system (Imaging Research, St. Catharines, ON, Canada).

### BBB Leakage Measurement

Cerebral infiltration of albuminuria, which is associated with DM, has been flagged as a predictor for cerebrovascular events (Anan et al., 2008) and as an index of BBB permeability (Wallin et al., 2000). In this study, to test whether L-4F treatment enhances BBB-integrity after stroke in T2DM mice, the albumin density in the ischemic brain was measured. The positive area of albumin in the ischemic border area is presented.

### Vascular Density, Perimeter/Diameter, and Cell Number Measurement

vWF-immunoreactivity was employed to identify vascular vessels, and αSMA was used as a marker of arterioles (Ho et al., 2006). The density and perimeter of vWF-coated vessels, and the number and diameter of αSMA-positive arterioles (have only one to two layers of SMCs, diameter 10–20 μm) were analyzed in the IBZ. The diameter is presented as the average of a total 10 largest arteries in the IBZ.

### WM Density and OPC Number Measurement

For measurement of WM-density, the BS, LFB, or SMI31 -positive area in the WM bundles of striatum and the total number of PDGFRα^+^-OPCs in the IBZ of the striatum were measured in each referenced coronal section using the MCID computer imaging analysis system.

### Real-Time RT-PCR Assay

Tissues from the ischemic area of the ipsilateral hemisphere from both vehicle-control and L-4F-treatment T2DM mice were isolated 4 days after MCAo. Total RNA was isolated using a standard protocol. Quantitative PCR was performed on an ABI 7000 PCR instrument (Applied Biosystems, Foster City, CA, United States) using three-stage program parameters provided by the manufacturer. Each sample was tested in triplicate, and analysis of relative gene expression data using the 2^–ΔΔ*CT*^ method. The following primers for RT-PCR were designed using Primer Express software (ABI). TNFα: Fwd: TACTCCCAGGTTCTCTTCAAGG, Rev: GAGGTTGACTTTCTCCTGGTA; PAI-1: Fwd: GTCTTTCCG ACCAAGAGCAG.

Rev: ATCACTTGGCCCATGAAGAG; GAPDH: Fwd: AGA ACA TCA TCC CTG CAT CC, Rev: CAC ATT GGG GGT AGG AAC AC.

### Western Blot (WB) Assay

Equal amounts of brain-tissue lysate from the ischemic area of the ipsilateral hemisphere were subjected to WB analysis. Specific proteins were visualized using a SuperSignal West Pico chemiluminescence kit (Pierce). The following primary antibodies were used: anti-RAGE (1:1000, rat, R&D MAB1179), anti-HMGB1 (1:1000, rabbit, Abcam ab18256), anti-TNFα (1: 1000, Abcam ab9755), anti-PAI-1 (1:500, rabbit, Santa Cruz sc-8979), anti-ED1 (CD68, 1:800, Bio-rad, MCA341, a single chain glycoprotein of 90–110 kDa, is the widely used pan-macrophage marker), and anti-β-actin (1:2000; Santa Cruz sc-1616) for 16 h at 4°C. The membranes were washed with blocking buffer without milk, and then incubated with horseradish peroxidase-conjugated secondary antibody in blocking buffer.

### Mouse Brain Cerebral Endothelial Cell (bEnd.3) Culture and Capillary-Like Tube Formation Assay

The bEnd.3 cell line (ATCC, cat# CRL02299) was used. Briefly, 0.1 ml growth factor reduced Matrigel (Becton Dickinson) was added per well of a 96 well plate, and cells (2 × 10^4^ cells) were seeded in Matrigel and cultured with serum free DMEM medium. The cells were divided into four groups (*n* = 6 wells/group), and treated with: (1) normal-glucose (12.5 mM glucose) as control; (2) high-glucose (HG, 37.5 mM glucose); (3) HG + L-4F (50 ng/ml); (4) HG + L-4F (100 ng/ml). Cells were incubated for 5 h and the capillary tube formation measurement was performed. For quantitative measurements of capillary tube formation, Matrigel wells were digitized under a 4X objective (Olympus BX40). Tracks of cells organized into networks of cellular cords (tubes) were counted and averaged in randomly selected three microscopic fields, and the total tube length of capillary tube formation was measured using a video camera (Sony DXC-970MD) interfaced with the MCID image analysis system.

### Primary Artery Cell Migration Measurement

To investigate whether L-4F treatment promotes arterial cell migration in hyperglyceridemia, a primary artery explant culture model was employed (Cui et al., 2011). The aorta was surgically removed from adult C57BL/6 wild-type mice (6–8 weeks, purchased from Jaxson Lab) 24 h after MCAo. The artery explant was cut to 1 mm^3^ and placed in Matrigel and cultured with DMEM medium containing 2% B27. The artery explants were divided into four groups as follows: (1) normal glucose (12.5 mM) as a control; (2) HG (37.5 mM glucose); (3) HG + L-4F (50 ng/ml); (4) HG + L-4F (100 ng/ml). Arterial explant cultures were allowed to grow for 5 days before being photographed and the 10 longest distances of outgrowth were measured under a microscope at 4X magnification, processed with the MCID and averaged. *n* = 6/group.

### Primary Cortical Neuron (PCN) Culture and Neurite/Axonal Outgrowth Measurements

The PCNs derived from E15 C57BL/6 embryos were employed, as previously described (Cui et al., 2016). Briefly, embryos were removed, and the cerebral cortex was dissected out, stripped of meninges, and dissociated by a combination of Ca^2+^- and Mg^2+^- free Hanks balance salt solution (HBSS) containing 0.125% trypsin digestion and mechanical trituration. The dissociated cell suspensions were seeded into poly-L-lysine precoated plates in a density of 10^5^ cells/cm^2^. The cells were grown in Dulbecco’s Modified Eagle Medium (DMEM, Invitrogen, Carlsbad, CA, United States) supplemented with 5% fetal bovine serum (FBS, GIBCO, Grand Island, NY, United States) at 37°C with 5% CO_2_. After 24 h, the cell cultures were switched to incubate with serum-free neurobasal medium (Invitrogen) with 2% B27 supplement (GIBCO), 0.5 mM L-glutamine, and 1% antibiotic- antimycotic media.

To test whether L-4F dose-dependently increases neurite-outgrowth in hyperglycemia after ischemia, the PCN cultures were subjected to 2 h of oxygen and glucose deprivation (OGD) on day-*in vitro* 4 (DIV4) followed by 24 h of reperfusion. The DIV5 OGD-PCNs were randomly divided into (*n* = 6 wells/group): (1) normal glucose control; (2) HG; (3) + L-4F 50 ng/ml; (4) + L-4F 100 ng/ml for 24 h. The PCN cultures were then stained with TUJ1 (a phenotypic marker of neural cells, 1:1000, Covance) with Cy3 and photographed using a 10 × objective fluorescent microscope (Zeiss). The average length of the 20 longest neurites in each well was calculated (Cui et al., 2016).

For testing whether L-4F increases axonal-outgrowth in HG condition, the axonal-outgrowth was measured using a microfluidic axonal growth model (Standard Neuron Device; catalog No SND450, Xona Microfluidics) (Cui et al., 2016). On DIV4, the PCN cultures were divided into four groups (*n* = 6 well/group): (1) normal glucose control; (2) HG; (3) + L-4F 50 ng/ml; (4) + L-4F 100 ng/ml. All axon-cultures were allowed to grow for an additional 48 h and were subjected to TUJ-1 immunostaining, and the axonal-outgrowth was measured on DIV6. The average length of the 10 longest axons in each well was calculated.

### Statistical Analysis

One-way ANOVA followed by Tukey *Post Hoc* Test were used for analysis of the differences among the three or four groups of sham, MCAo and L-4F treatment *in vivo* or *in vitro* experiments. *p* < 0.05 was set as a significant difference, and all data are presented as mean ± Standard Error (SE).

## Results

### L-4F-Treatment Deceases Hemorrhage/Lesion Volume and Mortality Rate, and Improves Functional Outcome After Stroke in T2DM Mice

**FIGURE 1:**
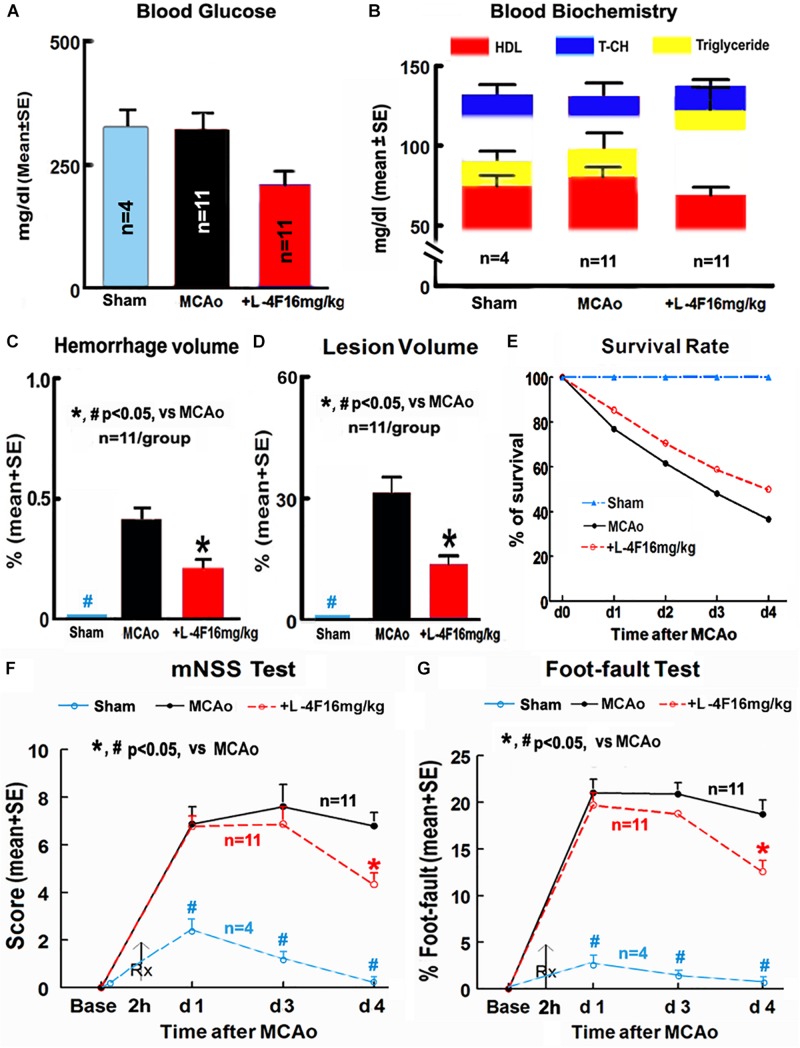
L-4F treatment did not change blood biochemistry, but decreases hemorrhage/lesion volumes in the ischemic brain and reduces mortality, and improves functional outcome in T2DM mice 4 days after MCAo. **(A)** Blood levels of glucose; **(B)** blood level of HDL, T-CH, and triglyceride; **(C)** hemorrhage volume; **(D)** lesion volumes; **(E)** survival rate; **(F,G)** functional outcome.

There were no significant differences in the levels of blood glucose, HDL, T-CH, and triglyceride among animals of sham-control (*n* = 4) and MCAo groups treated with or without L-4F (*n* = 11/group) 4 days after MCAo, respectively (Figures 1A,B). However, L-4F treatment of MCAo mice significantly decreased hemorrhage volume (Figure 1C, *n* = 11/group), and reduced lesion volume (Figure 1D, *n* = 11/group) and mortality rate (Figure 1E, *n* = 52 in MCAo-control group, *n* = 34 in L-4F treatment group) compared to the MCAo-control group (*p* < 0.05).

Stroke induced significant functional deficits as indicated by significantly increased mNSS and left foot-fault at 1, 3, and 4 days in T2DM mice in the MCAo-control group (*n* = 11) compared with non-stroke T2DM mice in the sham-control group (*n* = 4, *p* < 0.05). L-4F treatment significantly improved neurological functional outcome 4 days after MCAo in T2DM-stroke mice (Figures 1F,G, *p* < 0.05, *n* = 11/group). These data indicate that L-4F significantly decreases cerebral hemorrhagic transformation and reduces lesion volume and mortality as well as improves neurological functional outcome, independently of blood HDL and glucose level.

### L-4F Treatment Reduces Vascular Damage in the Ischemic Brain in T2DM Mice

**FIGURE 2:**
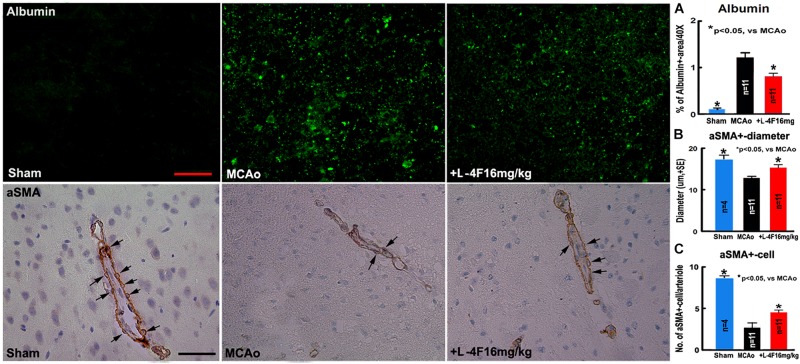
L-4F treatment decreases BBB-leakage, increases SMC number and arteriolar diameter in the ischemic brain in T2DM mice 4 days after MCAo; **(A)** albumin-immunostaining and quantitative data; **(B)** αSMA-immunostaining and quantitative data. **(C)** Numbers of αSMA^+^-cells in the arteriolar walls. Scale bar = 25 μm; *n* = 4 in sham-control group, *n* = 11 in MCAo-control and L-4F treatment group.

There was no albumin infiltration in the non-stroke brains (*n* = 4). However, albumin infiltration was observed in the ischemic core area in the T2DM-stroke brains, and the albumin density was significantly decreased in the L-4F treatment group compared with MCAo-control group (Figure 2A, *p* < 0.05, *n* = 11/group). These data indicate that the integrity of the BBB was compromised in the ischemic brain in T2DM stroke mice, and early L-4F treatment protects BBB integrity in the ischemic brain after stroke in T2DM mice. These data (i.e., BBB disruption as measured using albumin) are consistent with our previous study that D-4F enhances BBB-integrity as detected by Evans blue (EB) dye infiltration detection in the ischemic brain in C57BL/6 wild type mice after stroke (Cui et al., 2016).

Using vascular density, diameter and perimeter to indicate vascular structure changes, we found that there is no difference in the density/perimeter of vWF^+^-vessels among the sham-control, MCAo-control and L-4F treatment groups, and no differences in the density of αSMA^+^-arterioles between sham-control and MCAo-control groups. However, both the diameter of αSMA^+^-arterioles and the number of αSMA^+^-SMCs were significantly decreased in the MCAo-control group (*n* = 11) compared with sham-control group (*n* = 4), and the diameter of αSMA^+^-arterioles (Figure 2B) and the number of αSMA^+^-SMCs (Figure 2C) in the IBZ significantly increased in the L-4F treatment group compared with MCAo-control group (*p* < 0.05, *n* = 11/group). These data indicate that vascular structure was damaged in ischemic brain in T2DM stroke mice, and early L-4F treatment has a vascular protective effect on the ischemic brain after stroke in T2DM mice.

### L-4F Treatment Decreases WM-Damage and OPC Loss in the Ischemic Brain in T2DM Mice

**FIGURE 3:**
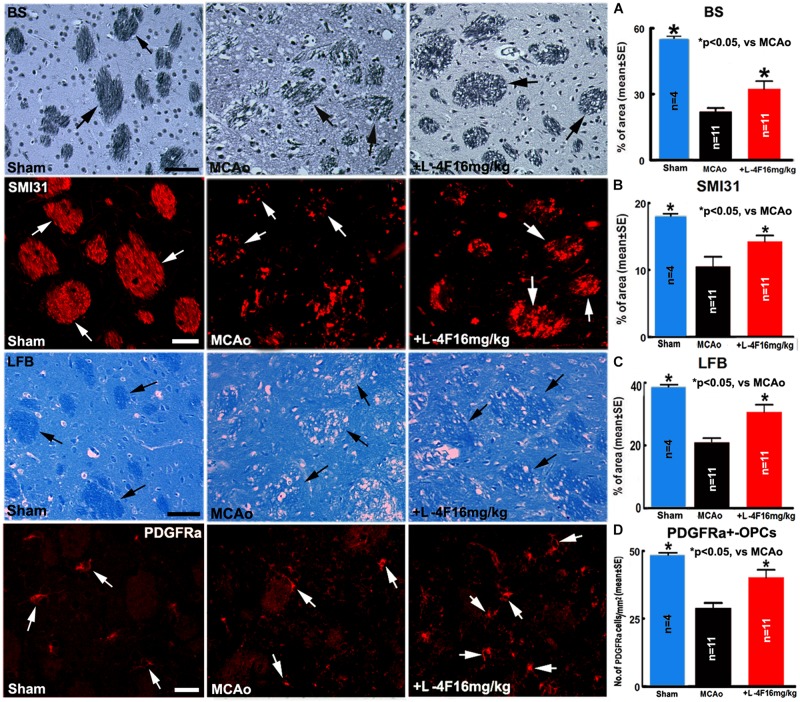
L-4F treatment increases WM density and OPC numbers in the ischemic brain in T2DM mice 4 days after stroke. **(A)** BS^+^ -axon and quantitative data; **(B)** SMI31^+^ -neurofilament and quantitative data; **(C)** LFB^+^ -myelin and quantitative data; **(D)** PDGFRα^+^ -OPCs and quantitative data. Scale bar in **(A,C)** = 40 μm; in **(B,D)** = 20 μm. ^∗^*p* < 0.05, *n* = 4 in sham-control group, *n* = 11 in MCAo-control and L-4F treatment group.

To investigate whether L-4F reduces WM-damage and OPC loss after stroke, we measured the densities of BS^+^ (axon marker, Figure 3A), αSMI31^+^ (phosphorylated-neurofilament marker, Figure 3B), LFB^+^ (myelin marker, Figure 3C) and the number of PDGFR(+-OPCs (Figure 3D) in the IBZ of striatum in the ischemic ipsilateral hemisphere, respectively. Compared with sham-control mice (*n* = 4), the MCAo-control mice (*n* = 11) exhibited a significant decrease in the densities of axons, phosphorylated-neurofilament, myelin and the number of OPCs 4 days after MCAo (*p* < 0.05). However, L-4F treatment significantly increased the densities of axons, phosphorylated-neurofilaments, myelin and the number of OPCs 4 days after MCAo compared with MCAo-control group (*p* < 0.05, *n* = 11/group). These data indicate that L-4F treatment decreases WM damage and OPC loss after stroke in T2DM mice.

### L-4F Treatment Reduces Macrophage Infiltration and Decreases RAGE/HMGB1 and PAI-1 Expression in the Ischemic Brain After Stroke in T2DM Mice

**FIGURE 4:**
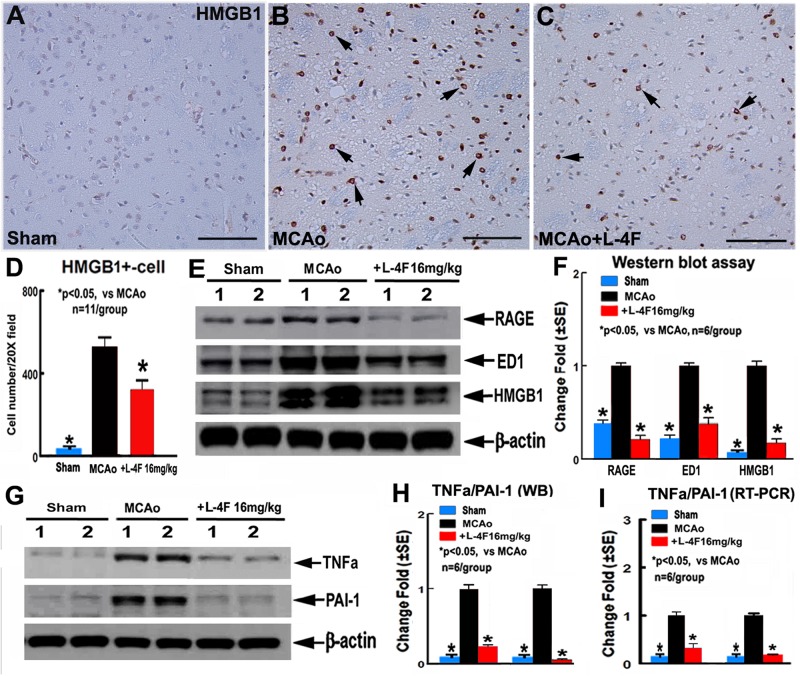
L-4F treatment decreases RAGE/ED1/HMGB-1, and PAI-1 in the ischemic brain in T2DM mice 4 days after MCAo. **(A–D)** HMGB-1 immunostaining and quantitative data; **(E,F)** RAGE/ED1/HMGB-1 WB assay and quantitative data; **(G,H)** TNFα/PAI-1 WB assay and quantitative data; **(I)** TNFα/PAI-1 RT-PCR data. Scale bar in **(A–C)** = 50 μm; ^∗^*p* < 0.05, *n* = 11/group in **(A–D)**; *n* = 6/group in **(A–I)**.

To investigate the mechanism underlying L-4F treatment-induced neuroprotection in T2DM, the macrophage infiltration was measured by ED1 protein level, and the protein and mRNA levels of HMGB1, RAGE, TNFα, and PAI-1 were measured by using immunostaining, WB or RT-PCR assay, respectively. The data show that L-4F treatment significantly decreases HMGB1 protein levels measured by immunostaining (Figures 4A–D, *n* = 11/group) and reduces RAGE/ED/HMGB1 level measured by WB (Figures 4E,F, *n* = 6/group). Moreover, L-4F treatment significantly decreases TNFα and PAI-1 protein and mRNA level (Figures 4G–I) in the ischemic ipsilateral brain compared to MCAo-control group (*p* < 0.05, *n* = 6/group).

### L-4F Treatment Does Not Change Capillary-Like Tube Formation, but Increases Artery Explant Cell Migration Under HG Condition

**FIGURE 5:**
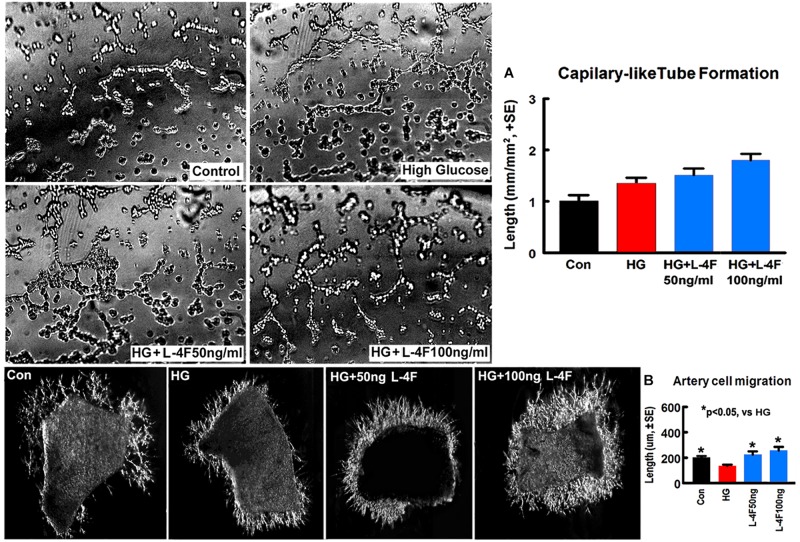
L-4F treatment does not change capillary-like tube formation, but increases artery cell migration in normal and HG condition. **(A)** *In vitro* capillary-like tube formation and quantitative data; **(B)** primary cultured artery cell migration and quantitative data in normal and high glucose condition. ^∗^*p* < 0.05, *n* = 6 well/group.

To confirm the *in vivo* findings, *in vitro* capillary-like tube formation assay for angiogenesis and primary artery explant cell migration for artery function test were employed. L-4F treatment at concentrations of 50 or 100 ng/ml did not increase capillary tube formation under HG conditions compared to non-treatment control (Figure 5A). However, L-4F treatment significantly increases artery explant cell migration at both 50 and 100 ng/ml concentration in HG media (Figure 5B, *p* < 0.05, *n* = 6/group) compared to non-treatment control, respectively. These data suggest that L-4F does not change angiogenesis, but increases arterial function in HG condition.

### L-4F Treatment Increases Neurite/Axonal Outgrowth in HG Condition

**FIGURE 6:**
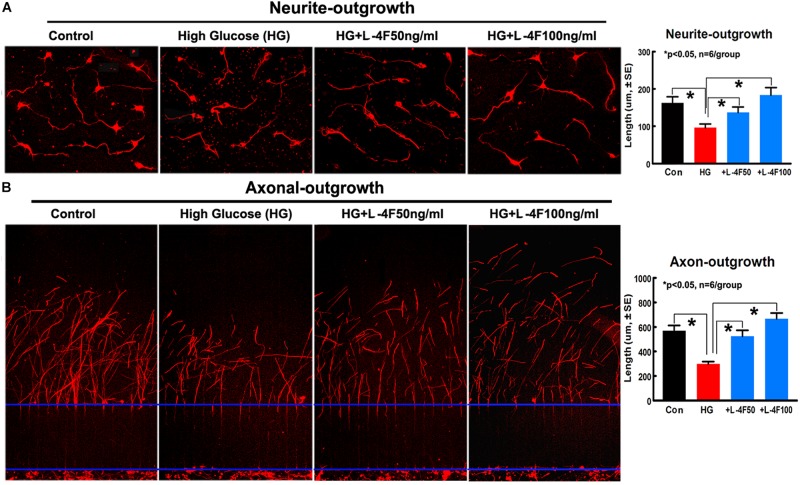
L-4F treatment increases neurite and axonal outgrowth under HG condition. **(A)** *In vitro* neurite outgrowth and quantitative data. **(B)** Axonal-outgrowth and quantitative data. ^∗^*p* < 0.05, *n* = 6 well/group.

To confirm the *in vivo* findings that L-4F treatment protects WM damage, we measured the neurite and axonal outgrowth under HG conditions. To mimic *in vivo* ischemia, the PCNs were subjected to 2 h of OGD. The data show that HG decreases PCN neurite outgrowth, but L-4F treatment (both 50 and 100 ng/ml) significantly increases PCN neurite outgrowth after ischemia (Figure 6A). In addition, L-4F (50 and 100 ng/ml) also increases and axonal outgrowth in HG condition compared with non-treatment control group (Figure 6B, *p* < 0.05, *n* = 6 wells/group).

## Discussion

T2DM confers increased micro- and macro- vascular complications and amplifies of morbidity and mortality after ischemic stroke (Basu et al., 2005; Madonna and De Caterina, 2011). Compared to non-DM stroke patients, T2DM stroke patients have chronic states of oxidative stress and inflammation, which accompany long-term endothelial dysfunction and impaired vasodilation, and increased vasogenic edema, BBB damage, and secondary hemorrhage transformation (Capes et al., 2001; Kruger et al., 2005; Ergul et al., 2007; Peterson et al., 2007; Ding et al., 2015). WM-damage is an important prognostic factor for the development of functional deficits after stroke (Anan et al., 2008), BBB and WM damage which are associated with enlarged infarct volume and worse functional outcome after stroke is found in both the experimental T2DM rodents and T2DM patients (Mooradian et al., 2005; Ennis and Keep, 2007; Anan et al., 2010). D-4F increases arterial concentrations of hemeoxygenase-1 and superoxide dismutase, decreases superoxide levels and endothelial cell fragmentation, and restores arterial vasoreactivity to normal in DM animal models (Ou et al., 2003, 2005; Kruger et al., 2005; Sherman et al., 2010). In a mouse model of systemic sclerosis, D-4F functioned to improve vasodilation and angiogenic potential, while reducing myocardial inflammation and oxidative stress (Sherman et al., 2010). In addition, L-4F dramatically improves impaired vasodilation in LDL receptor knockout (Ldlr−/−) mice, via decreased oxidative damage to endothelium (Ou et al., 2003). In our previous study, using a dose-dependent effect on MCAo model in wild type mice, an elevated concentration of D-4F (32 mg/kg) did not provide increased benefit on neurovascular and WM remodeling compared to treatment with a reduced concentration (16 mg/kg) (Cui et al., 2016). D-4F and L-4F have similar anti-inflammatory properties (Van Lenten et al., 2008; Liu et al., 2014; Yao et al., 2015). Therefore, in the present study, the dose of 16 mg/kg L-4F, the same as the optimal dose of D-4F identified from our previous study was used. db/db mice are gene knockout mice which mimic features of human T2DM and have been widely used to investigate diabetic stroke (Vannucci et al., 2001; Kumari et al., 2010; Chen et al., 2011; Cui et al., 2011; Kumari et al., 2011; Sims-Robinson et al., 2012). The db/db stroke mice exhibit increased neurovascular and WM damage and hemorrhagic/mortality rate compared with non-DM stroke mice (Chen et al., 2011; Cui et al., 2011; Ye et al., 2011). In the current study, db/db mice were employed as the T2DM model. We found that L-4F treatment initiated from 2 h post-stroke onset significantly decreased animal mortality rate and hemorrhage/infarct volume, decreased BBB leakage and WM damage, as well as increased vascular SMC and OPC numbers and enlarged arteriolar diameter in the ischemic brain 4 days after stroke. *In vitro* study shows that L-4F treatment did not change angiogenesis of mouse cerebral endothelial cells, but increased artery explant cell migration and neurite/axon outgrowth under HG condition. These data suggest that improving vascular integrity and retaining vasodilation as well as protecting WM damage, but not necessarily angiogenesis may play important role in L-4F induced neuroprotective effects.

In the brain of both T2DM patients and animal models, the extensive vascular and WM damage are highly associated with and are a consequence of inflammation (Golay et al., 1987; Williams and Nadler, 2007; Anan et al., 2010; Doll et al., 2015). Under normal physiology, RAGE is expressed at low levels, but it is highly upregulated in T2DM under chronic inflammation states because of the accumulation of various RAGE ligands such as AGEs and HMGB1. Blocking RAGE signaling in cell and animal models has revealed that targeting RAGE impairs inflammation and progression of DM vascular complications (Hudson and Lippman, 2018). M1-phage macrophages (ED1^+^) constitute about 70% of infiltrating cells and exert a major inflammatory function in the brain lesions (Tran et al., 1998). HMGB1 is mainly released from activated immune cells, such as the peripherally infiltrating immune cells (e.g., macrophage/monocytes) in the ischemic brain (Wang et al., 2010; Qin and Crews, 2012; Crews and Vetreno, 2014). HMGB-1 functions also as a proinflammatory factor, promotes cell toxicity and cell death, regulates clot-promoting properties which propagate further inflammation and coagulation (Semeraro et al., 2012), and contributes to the initiation and progression of stroke (Hu et al., 2016). In the present study, L-4F treatment significantly reduced macrophage infiltration and HMGB1/RAGE level. These data suggest that L-4F treatment suppresses neuroinflammation in the ischemic brain, which may contribute to L-4F induced neuroprotective effects in T2DM stroke mice.

High stroke risk in T2DM patients is often related to their accelerated endothelial dysfunction which is accompanied by an array of abnormalities, including altered endothelial-dependent vasodilation, and an imbalance between local pro- and anticlotting factors (Scarabin et al., 1998; Bonora, 2006; Al-Hamodi et al., 2011; Costa and Soares, 2013; Gorska-Ciebiada et al., 2016). The AGE/RAGE signaling pathway not only promotes inflammation such as TNFα and cell death, but also increases endothelial activation biomarker PAI-1 expression in cultured endothelial cells (He et al., 2003; Gregorio et al., 2018). Moreover, TNFα also exerted a stimulatory effect on PAI-1 protein release and increased PAI-1 mRNA levels in T2DM (Birgel et al., 2000; de Carvalho et al., 2006). Recombinant human HMGB1 increases the secretion of PAI-1 and tissue plasminogen activator (tPA) in cultured human endothelial cells, and elicits proinflammatory responses of endothelial cells and may contribute to alterations in endothelial cell function in human inflammation (Fiuza et al., 2003). HMGB-1 can bind to both tPA and plasminogen, and enhance tPA-dependent fibrinolytic activity, while PAI-1 is a key negative regulator of fibrinolysis through inhibition of plasminogen and tPA (Scarabin et al., 1998; Bonora, 2006; Al-Hamodi et al., 2011; Costa and Soares, 2013; Gorska-Ciebiada et al., 2016). Moreover, PAI-1 exerts various cellular effects independently of fibrinolysis, such as insulin resistance, metabolic abnormalities and endothelial dysfunction as well as pro-inflammatory effects (Cheng and Daskalakis, 2015; Kaji, 2016). PAI-1 is significantly increased in DM subjects compared with non-DM subjects with or without cardiovascular disease (Scarabin et al., 1998; Bonora, 2006; Al-Hamodi et al., 2011; Costa and Soares, 2013; Gorska-Ciebiada et al., 2016). In the present study, L-4F treatment significantly decreases TNFα and PAI-1 expression in the ischemic brain which may partially contribute to L-4F treatment-induced vascular and WM protection in T2DM stroke mice.

Plasma levels of HDL and glucose are highly associated with WM lesions in T2DM patients not receiving insulin treatment (Anan et al., 2010). Moreover, severe vascular and WM damage and neurological functional deficits in diabetic stroke populations are highly associated with dysfunction of HDL in the plasma (Anan et al., 2010; Kruit et al., 2010). Using ApoE-null (ApoE-/-) mice, administration of L-4F (20 mg/kg body weight, once daily subcutaneously) increases HDL level and ApoA-I concentration at 72 h post initial dosing (Chen et al., 2009). However in our study, T2DM mice subjected to stroke and treated with L-4F for 4 days did not exhibit significant changes in blood HDL cholesterol, triglyceride and glucose levels. Our data are consistent with others that D-4F or L-4F have no effect on blood T-CH or HDL concentrations (Navab et al., 2004, 2006, 2009; Kruger et al., 2005; Ou et al., 2005; Bloedon et al., 2008; Van Lenten et al., 2008; Yu et al., 2008; Morgantini et al., 2010; Sherman et al., 2010; Nandedkar et al., 2011). Other studies show that D-4F or L-4F reduces proinflammatory HDL levels and changes HDL function, increases cholesterol efflux, reduces lipoprotein oxidation and improves arterial vasoreactivity in T2DM patients, and in DM mice and rats (Navab et al., 2004, 2006, 2009; Kruger et al., 2005; Ou et al., 2005; Bloedon et al., 2008; Van Lenten et al., 2008; Yu et al., 2008; Morgantini et al., 2010; Sherman et al., 2010; Nandedkar et al., 2011). In addition, high levels of blood PAI-1 are associated with decreased functional HDL in T2DM patients (Bonora, 2006; Al-Hamodi et al., 2011; Gorska-Ciebiada et al., 2016). HDL can also regulate inflammatory responses in various types of cells that have been activated by proinflammatory stimuli in the arterial wall cells including endothelial cells and vascular SMCs (Mineo and Shaul, 2013). Therefore, we speculate that L-4F treatment may improve HDL function and decrease neuroinflammation, and thereby lessens vascular and WM damage after stroke in db/db T2DM mice.

## Conclusion

This study demonstrates that L-4F, an economical ApoA-I mimetic peptide, reduces neurovascular and WM damage via reducing proinflmammatory factors RAGE/HMGB-1 and TNFα/PAI-1 in the ischemic brain in db/db T2DM stroke mice. These provide a proof-of-concept that treatment with L-4F is a potential strategy to reduce neuroinflammation in the T2DM stroke population.

## Data Availability Statement

The raw data supporting the conclusions of this manuscript will be made available by the authors, without undue reservation, to any qualified researcher.

## Ethics Statement

Our animal use protocols were approved by the Guide for the Institute of Animal Care and Use Committee of the Henry Ford health System and in accordance with the standards of the Institutional Animal Care and Use Committee, National Institutes of Health. Adult male db/db mice (3 month old) were used.

## Author Contributions

XC, JC, and MC conceived and designed the study. XW, RL, AZ, JL-W, and XC acquisition of data and statistical analysis. XW and XC drafted the manuscript. JC and MC critical revision of the manuscript for intellectual content.

## Conflict of Interest

The authors declare that the research was conducted in the absence of any commercial or financial relationships that could be construed as a potential conflict of interest.

## References

[B1] Al-Hamodi Z. Ismail I. S. Saif-Ali R. Ahmed K. A. Muniandy S. (2011). Association of plasminogen activator inhibitor-1 and tissue plasminogen activator with type 2 diabetes and metabolic syndrome in Malaysian subjects. *Cardiovasc. Diabetol.* 10:23. 10.1186/1475-2840-10-23 21414238 PMC3064636

[B2] Anan F. Masaki T. Iwao T. Eto T. Shimomura T. Umeno Y. (2008). The role of microalbuminuria and insulin resistance as significant risk factors for white matter lesions in Japanese type 2 diabetic patients. *Curr. Med. Res. Opin.* 24 1561–1567. 10.1185/03007990802061818 18423105

[B3] Anan F. Masaki T. Kikuchi H. Iwao T. Shimomura T. Umeno Y. (2010). Association between plasma high-sensitivity C-reactive protein and insulin resistance and white matter lesions in Japanese type 2 diabetic patients. *Diabetes. Res. Clin. Pract.* 87 233–239. 10.1016/j.diabres.2009.10.017 19931932

[B4] Basu A. K. Pal S. K. Saha S. Bandyopadhyay R. Mukherjee S. C. Sarkar P. (2005). Risk factor analysis in ischaemic stroke: a hospital-based study. *J. Indian Med. Assoc.* 103 588. 16570759

[B5] Birgel M. Gottschling-Zeller H. Rohrig K. Hauner H. (2000). Role of cytokines in the regulation of plasminogen activator inhibitor-1 expression and secretion in newly differentiated subcutaneous human adipocytes. *Arterioscler. Thromb. Vasc. Biol.* 20 1682–1687. 10.1161/01.atv.20.6.1682 10845889

[B6] Bloedon L. T. Dunbar R. Duffy D. Pinell-Salles P. Norris R. DeGroot B. J. (2008). Safety, pharmacokinetics, and pharmacodynamics of oral apoA-I mimetic peptide D-4F in high-risk cardiovascular patients. *J. Lipid Res.* 49 1344–1352. 10.1194/jlr.P800003-JLR200 18323573 PMC2386905

[B7] Bonora E. (2006). The metabolic syndrome and cardiovascular disease. *Ann. Med.* 38 64–80. 16448990 10.1080/07853890500401234

[B8] Capes S. E. Hunt D. Malmberg K. Pathak P. Gerstein H. C. (2001). Stress hyperglycemia and prognosis of stroke in nondiabetic and diabetic patients: a systematic overview. *Stroke* 32 2426–2432. 10.1161/hs1001.096194 11588337

[B9] Chen J. Cui X. Zacharek A. Cui Y. Roberts C. Chopp M. (2011). White matter damage and the effect of matrix metalloproteinases in type 2 diabetic mice after stroke. *Stroke* 42 445–452. 10.1161/STROKEAHA.110.596486 21193743 PMC3108495

[B10] Chen J. Sanberg P. R. Li Y. Wang L. Lu M. Willing A. E. (2001). Intravenous administration of human umbilical cord blood reduces behavioral deficits after stroke in rats. *Stroke* 32 2682–2688. 10.1161/hs1101.098367 11692034

[B11] Chen X. Burton C. Song X. McNamara L. Langella A. Cianetti S. (2009). An apoA-I mimetic peptide increases LCAT activity in mice through increasing HDL concentration. *Int. J. Biol. Sci.* 5 489–499. 10.7150/ijbs.5.489 19680471 PMC2726446

[B12] Cheng C. Daskalakis C. (2015). Association of adipokines with insulin resistance, microvascular dysfunction, and endothelial dysfunction in healthy young adults. *Mediators Inflamm.* 2015:594039. 10.1155/2015/594039 26549941 PMC4621345

[B13] Costa P. Z. Soares R. (2013). Neovascularization in diabetes and its complications. Unraveling the angiogenic paradox. *Life Sci.* 92 1037–1045. 10.1016/j.lfs.2013.04.001 23603139

[B14] Crews F. T. Vetreno R. P. (2014). Neuroimmune basis of alcoholic brain damage. *Int. Rev. Neurobiol.* 118 315–357. 10.1016/B978-0-12-801284-0.00010-5 25175868 PMC5765863

[B15] Cui X. Chopp M. Zacharek A. Cui C. Yan T. Ning R. (2016). D-4F decreases white matter damage after stroke in mice. *Stroke* 47 214–220. 10.1161/STROKEAHA.115.011046 26604250 PMC4702511

[B16] Cui X. Chopp M. Zacharek A. Ye X. Roberts C. Chen J. (2011). Angiopoietin/Tie2 pathway mediates type 2 diabetes induced vascular damage after cerebral stroke. *Neurobiol. Dis.* 43 285–292. 10.1016/j.nbd.2011.04.005 21515377 PMC3096677

[B17] de Carvalho M. H. Colaco A. L. Fortes Z. B. (2006). Cytokines, endothelial dysfunction, and insulin resistance. *Arq. Bras. Endocrinol. Metabol.* 50 304–312. 16767296 10.1590/s0004-27302006000200016

[B18] Ding G. Yan T. Chen J. Chopp M. Li L. Li Q. (2015). Persistent cerebrovascular damage after stroke in type two diabetic rats measured by magnetic resonance imaging. *Stroke* 46 507–512. 10.1161/STROKEAHA.114.007538 25523056 PMC4308544

[B19] Doll D. N. Hu H. Sun J. Lewis S. E. Simpkins J. W. Ren X. (2015). Mitochondrial crisis in cerebrovascular endothelial cells opens the blood-brain barrier. *Stroke* 46 1681–1689. 10.1161/STROKEAHA.115.009099 25922503 PMC4418219

[B20] Dullaart R. P. Annema W. de Boer J. F. Tietge U. J. (2012). Pancreatic beta-cell function relates positively to HDL functionality in well-controlled type 2 diabetes mellitus. *Atherosclerosis* 222 567–573. 10.1016/j.atherosclerosis.2012.03.037 22541874

[B21] Ennis S. R. Keep R. F. (2007). Effect of sustained-mild and transient-severe hyperglycemia on ischemia-induced blood-brain barrier opening. *J. Cereb. Blood Flow Metab.* 27 1573–1582. 10.1038/sj.jcbfm.9600454 17293843

[B22] Ergul A. Elgebaly M. M. Middlemore M. L. Li W. Elewa H. Switzer J. A. (2007). Increased hemorrhagic transformation and altered infarct size and localization after experimental stroke in a rat model type 2 diabetes. *BMC Neurol.* 7:33. 17937795 10.1186/1471-2377-7-33 PMC2098774

[B23] Fiuza C. Bustin M. Talwar S. Tropea M. Gerstenberger E. Shelhamer J. H. (2003). Inflammation-promoting activity of HMGB1 on human microvascular endothelial cells. *Blood* 101 2652–2660. 10.1182/blood-2002-05-1300 12456506

[B24] Golay A. Zech L. Shi M. Z. Chiou Y. A. Reaven G. M. Chen Y. D. (1987). High density lipoprotein (HDL) metabolism in noninsulin-dependent diabetes mellitus: measurement of HDL turnover using tritiated HDL. *J. Clin. Endocrinol. Metab.* 65 512–518. 10.1210/jcem-65-3-512 3114304

[B25] Gomez-Banoy N. Cuevas V. Higuita A. Aranzalez L. H. Mockus I. (2016). Soluble tumor necrosis factor receptor 1 is associated with diminished estimated glomerular filtration rate in colombian patients with type 2 diabetes. *J. Diabetes Complications* 30 852–857. 10.1016/j.jdiacomp.2016.03.015 27068267

[B26] Gorska-Ciebiada M. Saryusz-Wolska M. Borkowska A. Ciebiada M. Loba J. (2016). Plasma levels of thrombomodulin, plasminogen activator inhibitor-1 and fibrinogen in elderly, diabetic patients with depressive symptoms. *Aging Clin. Exp. Res. Oct* 28 843–851. 10.1007/s40520-015-0504-3 26613755 PMC5014884

[B27] Gotto A. M. Jr. Brinton E. A. (2004). Assessing low levels of high-density lipoprotein cholesterol as a risk factor in coronary heart disease: a working group report and update. *J. Am. Coll. Cardiol.* 43 717–724. 10.1016/j.jacc.2003.08.061 14998606

[B28] Gregorio P. C. Favretto G. Sassaki G. L. Cunha R. S. Becker-Finco A. Pecoits-Filho R. (2018). Sevelamer reduces endothelial inflammatory response to advanced glycation end products. *Clin. Kidney J.* 11 89–98. 10.1093/ckj/sfx074 29423208 PMC5798142

[B29] He G. Bruun J. M. Lihn A. S. Pedersen S. B. Richelsen B. (2003). Stimulation of PAI-1 and adipokines by glucose in human adipose tissue in vitro. *Biochem. Biophys. Res. Commun.* 310 878–883. 10.1016/j.bbrc.2003.09.091 14550286

[B30] Ho T. K. Rajkumar V. Black C. M. Abraham D. J. Baker D. M. (2006). Increased angiogenic response but deficient arteriolization and abnormal microvessel ultrastructure in critical leg ischaemia. *Br. J. Surg.* 93 1368–1376. 10.1002/bjs.5496 16952207

[B31] Hu J. Liu B. Zhao Q. Jin P. Hua F. Zhang Z. (2016). Bone marrow stromal cells inhibits HMGB1-mediated inflammation after stroke in type 2 diabetic rats. *Neuroscience* 324 11–19. 10.1016/j.neuroscience.2016.02.058 26946264

[B32] Hudson B. I. Lippman M. E. (2018). Targeting RAGE signaling in inflammatory disease. *Annu. Rev. Med.* 69 349–364. 10.1146/annurev-med-041316-085215 29106804

[B33] Igarashi M. Hirata A. Yamaguchi H. Jimbu Y. Tominaga M. (2008). Pioglitazone reduces atherogenic outcomes in type 2 diabetic patients. *J. Atheroscler. Thromb.* 15 34–40. 10.5551/jat.e528 18270461

[B34] Imaizumi S. Navab M. Morgantini C. Charles-Schoeman C. Su F. Gao F. (2011). Dysfunctional high-density lipoprotein and the potential of apolipoprotein A-1 mimetic peptides to normalize the composition and function of lipoproteins. *Circ. J.* 75 1533–1538. 10.1253/circj.cj-11-0460 21628835 PMC3625624

[B35] Kaji H. (2016). Adipose tissue-derived plasminogen activator inhibitor-1 function and regulation. *Compr Physiol.* 6 1873–1896. 10.1002/cphy.c160004 27783862

[B36] Kontush A. Chapman M. J. (2006). Functionally defective high-density lipoprotein: a new therapeutic target at the crossroads of dyslipidemia, inflammation, and atherosclerosis. *Pharmacol. Rev.* 58 342–374. 10.1124/pr.58.3.1 16968945

[B37] Kruger A. L. Peterson S. Turkseven S. Kaminski P. M. Zhang F. F. Quan S. (2005). D-4F induces heme oxygenase-1 and extracellular superoxide dismutase, decreases endothelial cell sloughing, and improves vascular reactivity in rat model of diabetes. *Circulation* 111 3126–3134. 10.1161/circulationaha.104.517102 15939814

[B38] Kruit J. K. Brunham L. R. Verchere C. B. Hayden M. R. (2010). HDL and LDL cholesterol significantly influence beta-cell function in type 2 diabetes mellitus. *Curr. Opin. Lipidol.* 21 178–185. 10.1097/MOL.0b013e328339387b 20463468

[B39] Kumari R. Willing L. B. Patel S. D. Baskerville K. A. Simpson I. A. (2011). Increased cerebral matrix metalloprotease-9 activity is associated with compromised recovery in the diabetic db/db mouse following a stroke. *J. Neurochem.* 119 1029–1040. 10.1111/j.1471-4159.2011.07487.x 21923664 PMC3217107

[B40] Kumari R. Willing L. B. Patel S. D. Krady J. K. Zavadoski W. J. Gibbs E. M. (2010). The PPAR-gamma agonist, darglitazone, restores acute inflammatory responses to cerebral hypoxia-ischemia in the diabetic ob/ob mouse. *J. Cereb. Blood Flow Metab.* 30 352–360. 10.1038/jcbfm.2009.221 19861974 PMC2949120

[B41] Liu J. Yao S. Wang S. Jiao P. Song G. Yu Y. (2014). D-4F, an apolipoprotein A-I mimetic peptide, protects human umbilical vein endothelial cells from oxidized low-density lipoprotein-induced injury by preventing the downregulation of pigment epithelium-derived factor expression. *J. Cardiovasc. Pharmacol.* 63 553–561. 10.1097/FJC.0000000000000080 24709637

[B42] Liu X. S. Zhang Z. G. Zhang R. L. Gregg S. R. Meng H. Chopp M. (2007). Comparison of in vivo and in vitro gene expression profiles in subventricular zone neural progenitor cells from the adult mouse after middle cerebral artery occlusion. *Neuroscience* 146 1053–1061. 10.1016/j.neuroscience.2007.02.056 17428613 PMC1942046

[B43] Madonna R. De Caterina R. (2011). Cellular and molecular mechanisms of vascular injury in diabetes–part I: pathways of vascular disease in diabetes. *Vasc. Pharmacol.* 54 68–74. 10.1016/j.vph.2011.03.005 21453786

[B44] Maron D. J. (2000). The epidemiology of low levels of high-density lipoprotein cholesterol in patients with and without coronary artery disease. *Am. J. Cardiol.* 86 11L–14L. 11374848 10.1016/s0002-9149(00)01462-4

[B45] Miller N. E. Olszewski W. L. Hattori H. Miller I. P. Kujiraoka T. Oka T. (2013). Lipoprotein remodeling generates lipid-poor apolipoprotein A-I particles in human interstitial fluid. *Am. J. Physiol. Endocrinol. Metab.* 304 E321–E328. 10.1152/ajpendo.00324.2012 23233540 PMC3566430

[B46] Mineo C. Shaul P. W. (2013). Regulation of signal transduction by HDL. *J. Lipid Res.* 54 2315–2324. 10.1194/jlr.R039479 23687307 PMC3735931

[B47] Mooradian A. D. Haas M. J. Batejko O. Hovsepyan M. Feman S. S. (2005). Statins ameliorate endothelial barrier permeability changes in the cerebral tissue of streptozotocin-induced diabetic rats. *Diabetes Metab. Res. Rev.* 54 2977–2982. 10.2337/diabetes.54.10.2977 16186401

[B48] Morgantini C. Imaizumi S. Grijalva V. Navab M. Fogelman A. M. Reddy S. T. (2010). Apolipoprotein A-I mimetic peptides prevent atherosclerosis development and reduce plaque inflammation in a murine model of diabetes. *Diabetes Metab. Res. Rev.* 59 3223–3228. 10.2337/db10-0844 20826564 PMC2992786

[B49] Mulder D. J. de Boer J. F. Graaff R. de Vries R. Annema W. Lefrandt J. D. (2011). Skin autofluorescence is inversely related to HDL anti-oxidative capacity in type 2 diabetes mellitus. *Atherosclerosis* 218 102–106. 10.1016/j.atherosclerosis.2011.05.011 21665206

[B50] Nandedkar S. D. Weihrauch D. Xu H. Shi Y. Feroah T. Hutchins W. (2011). D-4F, an apoA-1 mimetic, decreases airway hyperresponsiveness, inflammation, and oxidative stress in a murine model of asthma. *J. Lipid Res.* 52 499–508. 10.1194/jlr.M012724 21131532 PMC3035686

[B51] Navab M. Anantharamaiah G. M. Reddy S. T. Fogelman A. M. (2006). Apolipoprotein A-I mimetic peptides and their role in atherosclerosis prevention. *Nat. Clin. Pract. Cardiovasc. Med.* 3 540–547. 10.1038/ncpcardio0661 16990839

[B52] Navab M. Anantharamaiah G. M. Reddy S. T. Hama S. Hough G. Grijalva V. R. (2004). Oral D-4F causes formation of pre-beta high-density lipoprotein and improves high-density lipoprotein-mediated cholesterol efflux and reverse cholesterol transport from macrophages in apolipoprotein E-null mice. *Circulation* 109 3215–3220. 10.1161/01.cir.0000134275.90823.87 15197147

[B53] Navab M. Ruchala P. Waring A. J. Lehrer R. I. Hama S. Hough G. (2009). A novel method for oral delivery of apolipoprotein mimetic peptides synthesized from all L-amino acids. *J. Lipid Res.* 50 1538–1547. 10.1194/jlr.M800539-JLR200 19225094 PMC2724044

[B54] Nayak B. S. Ramsingh D. Gooding S. Legall G. Bissram S. Mohammed A. (2010). Plasma adiponectin levels are related to obesity, inflammation, blood lipids and insulin in type 2 diabetic and non-diabetic Trinidadians. *Prim. Care Diabetes* 4 187–192. 10.1016/j.pcd.2010.05.006 20580627

[B55] Ning R. Venkat P. Chopp M. Zacharek A. Yan T. Cui X. (2017). D-4F increases microRNA-124a and reduces neuroinflammation in diabetic stroke rats. *Oncotarget* 8 95481–95494. 10.18632/oncotarget.20751 29221142 PMC5707036

[B56] Ou J. Ou Z. Jones D. W. Holzhauer S. Hatoum O. A. Ackerman A. W. (2003). L-4F, an apolipoprotein A-1 mimetic, dramatically improves vasodilation in hypercholesterolemia and sickle cell disease. *Circulation* 107 2337–2341. 10.1161/01.cir.0000070589.61860.a9 12732610

[B57] Ou J. Wang J. Xu H. Ou Z. Sorci-Thomas M. G. Jones D. W. (2005). Effects of D-4F on vasodilation and vessel wall thickness in hypercholesterolemic LDL receptor-null and LDL receptor/apolipoprotein A-I double-knockout mice on Western diet. *Cir. Res.* 97 1190–1197. 10.1161/01.res.0000190634.60042.cb 16224061 PMC1480357

[B58] Peterson S. J. Husney D. Kruger A. L. Olszanecki R. Ricci F. Rodella L. F. (2007). Long-term treatment with the apolipoprotein A1 mimetic peptide increases antioxidants and vascular repair in type I diabetic rats. *J. Pharmacol. Exp. Ther.* 322 514–520. 10.1124/jpet.107.119479 17488882

[B59] Qin L. Crews F. T. (2012). Chronic ethanol increases systemic TLR3 agonist-induced neuroinflammation and neurodegeneration. *J. Neuroinflam.* 9:130. 10.1186/1742-2094-9-130 22709825 PMC3412752

[B60] Qin S. Kamanna V. S. Lai J. H. Liu T. Ganji S. H. Zhang L. (2012). Reverse D4F, an apolipoprotein-AI mimetic peptide, inhibits atherosclerosis in ApoE-null mice. *J. Cardiovasc. Pharmacol. Ther.* 17 334–343. 10.1177/1074248411434598 22308547

[B61] Scarabin P. Y. Aillaud M. F. Amouyel P. Evans A. Luc G. Ferrieres J. (1998). Associations of fibrinogen, factor VII and PAI-1 with baseline findings among 10,500 male participants in a prospective study of myocardial infarction–the PRIME Study. Prospective epidemiological study of myocardial infarction. *Thromb. Haemost.* 80 749–756. 10.1055/s-0037-1615353 9843166

[B62] Scheffer P. G. Tushuizen M. E. Vermue H. P. Schindhelm R. K. Rustemeijer C. Diamant M. (2011). Effect of three consecutive meals on the physicochemical properties of HDL and LDL in individuals with the metabolic syndrome and patients with type 2 diabetes. *Eur. J. Clin. Nutr.* 65 1242–1249. 10.1038/ejcn.2011.114 21712838

[B63] Semeraro N. Ammollo C. T. Semeraro F. Colucci M. (2012). Sepsis, thrombosis and organ dysfunction. *Thromb. Res.* 129 290–295. 10.1016/j.thromres.2011.10.013 22061311

[B64] Sharrett A. R. Ballantyne C. M. Coady S. A. Heiss G. Sorlie P. D. Catellier D. (2001). Coronary heart disease prediction from lipoprotein cholesterol levels, triglycerides, lipoprotein(a), apolipoproteins A-I and B, and HDL density subfractions: the atherosclerosis risk in communities (ARIC) study. *Circulation* 104 1108–1113. 10.1161/hc3501.095214 11535564

[B65] Shehadah A. Chen J. Pal A. He S. Zeitlin A. Cui X. (2014). Human placenta-derived adherent cell treatment of experimental stroke promotes functional recovery after stroke in young adult and older rats. *PLoS One* 9:e86621. 10.1371/journal.pone.0086621 24466174 PMC3897748

[B66] Sherman C. B. Peterson S. J. Frishman W. H. (2010). Apolipoprotein A-I mimetic peptides: a potential new therapy for the prevention of atherosclerosis. *Cardiol. Rev.* 18 141–147. 10.1097/CRD.0b013e3181c4b508 20395699

[B67] Sims-Robinson C. Zhao S. Hur J. Feldman E. L. (2012). Central nervous system endoplasmic reticulum stress in a murine model of type 2 diabetes. *Diabetologia* 55 2276–2284. 10.1007/s00125-012-2573-6 22581041 PMC3391332

[B68] Swanson R. A. Morton M. T. Tsao-Wu G. Savalos R. A. Davidson C. Sharp F. R. (1990). A semiautomated method for measuring brain infarct volume. *J. Cereb. Blood Flow Metab.* 10 290–293. 10.1038/jcbfm.1990.47 1689322

[B69] Tran E. H. Hoekstra K. van Rooijen N. Dijkstra C. D. Owens T. (1998). Immune invasion of the central nervous system parenchyma and experimental allergic encephalomyelitis, but not leukocyte extravasation from blood, are prevented in macrophage-depleted mice. *J. Immunol.* 161 3767–3775. 9759903

[B70] Van Lenten B. J. Wagner A. C. Jung C. L. Ruchala P. Waring A. J. Lehrer R. I. (2008). Anti-inflammatory apoA-I-mimetic peptides bind oxidized lipids with much higher affinity than human apoA-I. *J. Lipid Res.* 49 2302–2311. 10.1194/jlr.M800075-JLR200 18621920 PMC2563211

[B71] Vannucci S. J. Willing L. B. Goto S. Alkayed N. J. Brucklacher R. M. Wood T. L. (2001). Experimental stroke in the female diabetic, db/db, mouse. *J. Cereb. Blood Flow Metab.* 21 52–60. 10.1097/00004647-200101000-00007 11149668

[B72] Wallin A. Sjogren M. Edman A. Blennow K. Regland B. (2000). Symptoms, vascular risk factors and blood-brain barrier function in relation to CT white-matter changes in dementia. *Eur. Neurol.* 44 229–235. 10.1159/000008242 11096223

[B73] Wang H. Li W. Zhu S. Li J. D’Amore J. Ward M. F. (2010). Peripheral administration of fetuin-A attenuates early cerebral ischemic injury in rats. *J. Cereb. Blood Flow Metab.* 30 493–504. 10.1038/jcbfm.2009.247 19953099 PMC2860738

[B74] Williams M. D. Nadler J. L. (2007). Inflammatory mechanisms of diabetic complications. *Curr. Diab. Rep* 7 242–248. 10.1007/s11892-007-0038-y 17547842

[B75] Yao S. Tian H. Miao C. Zhang D. W. Zhao L. Li Y. (2015). D4F alleviates macrophage-derived foam cell apoptosis by inhibiting CD36 expression and ER stress-CHOP pathway. *J. Lipid Res.* 56 836–847. 10.1194/jlr.M055400 25635126 PMC4373741

[B76] Ye X. Chopp M. Liu X. Zacharek A. Cui X. Yan T. (2011). Niaspan reduces high-mobility group box 1/receptor for advanced glycation endproducts after stroke in type-1 diabetic rats. *Neuroscience* 190 339–345. 10.1016/j.neuroscience.2011.06.004 21683770 PMC3260534

[B77] Yu R. Yekta B. Vakili L. Gharavi N. Navab M. Marelli D. (2008). Proatherogenic high-density lipoprotein, vascular inflammation, and mimetic peptides. *Curr. Atheroscler. Rep.* 10 171–176. 10.1007/s11883-008-0025-z 18417073

